# Effect of Hibiscus rosa sinensis aqueous extract treatment on biochemical parameters in diabetic pregnant rats

**DOI:** 10.1186/1758-5996-7-S1-A77

**Published:** 2015-11-11

**Authors:** Thaís Leal Silva, Luana Alves Freitas Afiune, Rafaianne Queiroz de Moraes Souza, Thaigra de Sousa Soares, Thalita Bohnen Carneiro, Madilene Francely Américo, Kleber Eduardo de Campos, Gustavo Tadeu Volpato

**Affiliations:** 1 Universidade Federal de Mato Grosso, Barra do Garças, Brazil

## Background

The medications for diabetes treatment does not present with full efficient, so there is a search for new alternatives, one being the use of herbal medicines. Hibiscus rosa sinensis, commonly known as rose mallow, is widely used in Brazilian folk medicine for the diabetes treatment.

## Objective

To evaluate the effect of Hibiscus rosa sinensis aqueous extract treatment on biochemical parameters and oxidative stress in diabetic and non-diabetic pregnant rats.

## Materials and methods

Diabetes was induced by streptozotocin (40 mg/Kg) in virgin female Wistar rats. After diabetes induction, rats were mated. The pregnant diabetic rats were divided in four experimental groups (n minimum=12 animals/group): Non-diabetic; Non-diabetic Treated; Diabetic and Diabetic Treated. Oral administration of aqueous extract of Hibiscus rosa sinensis flowers was given to non-diabetic and diabetic pregnant rats at increasing doses: 100 mg/kg from day 0 to 7 of pregnancy, 200 mg/kg from day 8 to 14 and 400 mg/kg from day 15 to 20. On days 0, 7, 14 and 21 were measured glycaemia. On day 21 of pregnancy, all rats were anesthetized and killed, and the blood and liver were collected. The biochemical serum parameters (glycemic level, alanine aminotransferase [ALT], protein, cholesterol, triglycerides, High-density level lipoprotein [HDL]) and hepatic oxidative stress biomarkers (malondialdehyde [MDA], superoxide dismutase [SOD], catalase, total glutathione, thiol group) were analyze. Analysis of variance followed by Tukey's test was used. Differences were considered statistically significant when p< 0.05.

## Results

After treatment with Hibiscus rosa sinensis extract, non-diabetic and diabetic rats presented no glycemic changes. All the experimental groups showed decreasing in HDL levels compared to control group. Both diabetic groups presented higher ALT activity and MDA concentration. Also, the diabetic group presented high levels of triglycerides and total cholesterol compared to control group. The treatment with H. rosa sinensis in diabetic group was able to decrease the triglycerides and ALT levels compared to diabetic non-treated animals.

## Conclusion

The treatment with chalices of H. rosa sinensis aqueous extract showed no hypoglycemic effect and also did not alter other biochemical parameters and the stress oxidative biomarkers. However, this plant improves the biochemical parameters mainly in diabetic rats.

